# Supplementary material for: Navigating the credibility risks of environmental scientists’ activism
Source: Commun Psychol. 2026 Feb 21;4:61. doi: 10.1038/s44271-026-00409-8 (PMC13038406; doi:10.1038/s44271-026-00409-8)
Supplement: Supplementary file 2 — Supplementary Analyses [file 44271_2026_409_MOESM2_ESM.pdf]

Navigating the Credibility Risks of Environmental Scientists' Climate Activism

Supplementary

Study 1

Method

Participants and Design

The 222 incomplete entries and test-runs in our raw dataset were comprised as follows: Forty-four participants dropped out before consenting to the study, 99 before the first trait item of the first comment, 75 before the first trait item of the second comment, one before the moderator items, and three before the demographics.

Since participants were asked about the demographics at the end of our survey and the sample providers did not supply data about the demographics, we could not assess whether attrition was systematically related to any demographic variables. We calculated Chi²-tests to determine if attrition was related to our randomization. Neither target sex (between-subject factor), $\chi^2(1, N = 1267) = 0.77, p = .381$, Cramer's $V = .02$, nor the combination of person order, activist condition order, and topic order (within-subject factors), $\chi^2(7, N = 1267) = 0.91$, $p = .996$, Cramer's $V = .03$, showed a significant change in the distribution of the randomized assignments due to drop-outs.

Results

We used R[1] including the packages psych[2], readxl[3], stringr[4], dplyr[5], reshape2[6], effsize[7], interactions[8], lmerTest[9], jtools[10], ggplot2[11], gridExtra[12], gridGraphics[13], cowplot[14], broom[15], lme4[16], doSNOW[17], foreach[18], afex[19], effectsize[20], and emmeans[21] to analyze our data. We assessed scale reliability using Cronbach's α.

Descriptive Analyses and Counter-Balancing Checks

We observed no significant differences across Topics (Energy saving, Recycling), either for hypocrisy, $t(490) = -0.21, p = .832, d = 0.01$, 95% CI [-0.04, 0.07], or for competence, $t(490) = 0.91, p = .363, d = -0.05$, 95% CI [-0.13, 0.01]. Entering survey

Completion Time as a moderator yielded no significant Condition × Completion Time interactions: competence, $B < -0.01$, $SE < 0.01$, $t(489) = -0.25$, $p = .799$, and hypocrisy, $B < -0.01$, $SE < 0.01$, $t(489) = -0.45$, $p = .655$.

Moderation Analyses

On measures of competence, we observed significant Condition × Environmental Behaviour, $B = 0.15$, $SE = 0.04$, $t(489) = 3.69$, $p < .001$, Condition × Trust in Science, $B = 0.15$, $SE = 0.04$, $t(489) = 3.71$, $p < .001$, and Condition × Conspiracy Mentality interactions, $B = 0.10$, $SE = 0.04$, $t(489) = 2.71$, $p = .007$.

Condition effects on hypocrisy were not moderated by environmental behaviour, $B = -0.04$, $SE = 0.04$, $t(489) = -0.82$, $p = .415$, trust in science, $B = -0.06$, $SE = 0.04$, $t(489) = -1.51$, $p = .132$, or conspiracy mentality, $B = 0.04$, $SE = 0.04$, $t(489) = 1.15$, $p = .251$. We also observed no Condition × Reactance interactions on competence, $B = 0.03$, $SE = 0.04$, $t(489) = 0.79$, $p = .431$, or hypocrisy, $B = 0.05$, $SE = 0.04$, $t(489) = 1.32$, $p = .188$ or Condition × Resistance to Change interactions on competence, $B = 0.08$, $SE = 0.04$, $t(489) = 1.96$, $p = .051$, or hypocrisy, $B = 0.08$, $SE = 0.04$, $t(489) = 1.88$, $p = .060$.

Regarding demographics, a significant Condition × Age interaction emerged on hypocrisy, $B = 0.01$, $SE < 0.01$, $t(484) = 2.16$, $p = .032$, but not on competence, $B < -0.01$, $SE < 0.01$, $t(484) = -0.97$, $p = .335$. Johnson-Neyman analyses indicated that the condition effect was not significant for younger participants (< 39 years; 5 participants did not indicate their age). We did not observe systematic interactions of participant gender, target sex, or their combination with condition (5 participants did not indicate their gender; unfortunately, too few non-binary [$n = 2$] and diverse [$n = 1$] people participated to be included in this analysis): hypocrisy, Condition × Participant Gender, $F(1, 479) = 0.14$, $p = .709$, $\eta^2_p < .01$, 90% CI [<.01, .01], Condition × Target Sex, $F(1, 479) = 2.41$, $p = .121$, $\eta^2_p = .01$, 90% CI [<.01, .02], Condition × Participant Gender × Target Sex, $F(1, 479) = 4.76$, $p = .030$, $\eta^2_p = .01$, 90% CI [<.01, .03], and competence, Condition × Participant Gender, $F(1, 479) = 0.04$, $p = .845$, $\eta^2_p <$

.01, 90% CI [<.01, <.01], Condition × Target Sex, $F(1, 479) = 1.42$, $p = .234$, $\eta^2_p < .01$, 90% CI [<.01, .02], Condition × Participant Gender × Target Sex, $F(1, 479) = 0.31$, $p = .577$, $\eta^2_p <$ .01, 90% CI [<.01, .01].

Including participants' self-description as an activist (yes vs. no) as an exploratory factor showed a significant Source Activism × Participant Activism interaction on competence, $F(1, 481) = 13.46$, $p < .001$, $\eta^2_p = .03$, 90% CI [.01, .06]. Follow-up analyses (Bonferroni-corrected) indicated that the message source effect on competence was significant among non-activist participants, $t(481) = 5.41$, $p < .001$, but not among activist participants, $t(481) = 0.64$, $p > .999$. No parallel interaction was observed on hypocrisy, $F(1, 481) = 0.84$, $p = .359$, $\eta^2_p < .01$, 90% CI [<.01, .01]. Turning to participants' self-description as a scientist (yes vs. no) as an exploratory factor, we did not observe a significant interaction with Source Activism on either competence, $F(1, 481) = 1.88$, $p = .171$, $\eta^2_p < .01$, 90% CI [<.01, .02], or hypocrisy, $F(1, 481) = 0.37$, $p = .544$, $\eta^2_p < .01$, 90% CI [<.01, .01].

Study 2

Method

Participants and Design

The 129 incomplete entries and test-runs in our raw dataset were comprised as follows: Ninety-five dropped out before consenting to the study, 18 before the moderator items (i.e., during or immediately after the demographics), 9 before the first trait item of the first comment, and 7 before the first trait item of the second comment. We performed attrition analyses using Wilcoxon rank sum tests to compare the characteristics of participants who dropped out after providing demographics to participants who completed the study. We observed no reliable differences between the two groups in terms of political orientation, $W = 2805.50$, $p = .351$, income, $W = 1459.50$, $p = .087$, and SES, $W = 2530.50$, $p = .699$.

Results

Descriptive Analyses and Counter-Balancing Checks

We observed no significant differences across Topics (Energy consumption, Consumer products) for any of the dependent measures: hypocrisy, $t(635) < -0.01$, $p = .997$, $d < 0.01$, 95% CI [-0.09, 0.09], expertise-based trust, $t(635) = 0.24$, $p = .811$, $d = -0.01$, 95% CI [-0.10, 0.08], integrity-based trust, $t(635) = 0.19$, $p = .846$, $d = -0.01$, 95% CI [-0.10, 0.08], benevolence-based trust, $t(635) = 0.02$, $p = .980$, $d < -0.01$, 95% CI [-0.10, 0.10], researcher's credibility, $t(635) = -0.26$, $p = .794$, $d = 0.01$, 95% CI [-0.07, 0.10], action intent, $t(635) = 0.37$, $p = .714$, $d = -0.02$, 95% CI [-0.08, 0.04], attribution of environmental concern, $t(635) = 0.26$, $p = .796$, $d = -0.01$, 95% CI [-0.09, 0.06], and trust in the researchers' field, $t(635) = 0.40$, $p = .684$, $d = -0.02$, 95% CI [-0.09, 0.04].

Entering survey Completion Time as a moderator yielded no significant Condition × Completion Time interaction on hypocrisy, $B < 0.01$, $SE < 0.01$, $t(634) = 0.39$, $p = .698$, expertise-based trust, $B < 0.01$, $SE < 0.01$, $t(634) = 0.12$, $p = .906$, integrity-based trust, $B < 0.01$, $SE < 0.01$, $t(634) = 0.26$, $p = .795$, benevolence-based trust, $B < 0.01$, $SE < 0.01$, $t(634) = 0.29$, $p = .775$, researcher's credibility, $B < 0.01$, $SE < 0.01$, $t(634) = 0.35$, $p = .728$, action intent, $B < 0.01$, $SE < 0.01$, $t(634) = 0.58$, $p = .564$, attribution of environmental concern, $B < 0.01$, $SE < 0.01$, $t(634) = 1.70$, $p = .091$, and trust in the researchers' field, $B < 0.01$, $SE < 0.01$, $t(634) = 0.26$, $p = .792$.

Moderation Analyses

We explored if the expected (i.e., pre-registered) effects were moderated by participants' own pro-environmental behaviour, conspiracy mentality, trust in science, or demographics (political orientation, age, gender). We used a corrected $\alpha = .017$.

We observed significant Condition × Environmental Behaviour interactions on all dependent measures: expertise-based trust, $B = 0.12$, $SE = 0.04$, $t(634) = 3.03$, $p = .003$, credibility, $B = 0.27$, $SE = 0.07$, $t(634) = 3.72$, $p < .001$, hypocrisy, $B = -0.18$, $SE = 0.04$, $t(634) = -4.43$, $p < .001$, and trust in the researchers' field, $B = 0.15$, $SE = 0.04$, $t(634) = 4.15$, $p < .001$. We also observed consistent Condition × Trust in Science interactions on all

dependent measures: expertise-based trust, $B = 0.17$, $SE = 0.04$, $t(634) = 4.07$, $p < .001$, hypocrisy, $B = -0.20$, $SE = 0.04$, $t(634) = -4.78$, $p < .001$, credibility, $B = 0.34$, $SE = 0.07$, $t(634) = 4.55$, $p < .001$, and trust in the researchers' field, $B = 0.16$, $SE = 0.04$, $t(634) = 4.11$, $p < .001$. We observed no significant Condition × Conspiracy Mentality interactions on any of the dependent measures: Expertise-based trust, $B = -0.07$, $SE = 0.04$, $t(634) = -2.03$, $p = .043$, credibility, $B = -0.07$, $SE = 0.06$, $t(634) = -1.01$, $p = .311$, hypocrisy, $B = 0.05$, $SE = 0.04$, $t(634) = 1.44$, $p = .151$, and trust in the researchers' field, $B < -0.01$, $SE = 0.03$, $t(634) = -0.07$, $p = .948$.

Johnson-Neyman analyses indicated that participants engaging in more pro-environmental behaviour, exhibiting lower conspiracy mentality, and with higher trust in science showed smaller negative effects of scientists' activism. Significant transition points emerged such that the condition effect was non-significant on trust in the researchers' field for participants high in pro-environmental behaviour (scale value above 5.96) and trust in science (scale value above 6.64; Supplementary Fig. 1). No other transition points were observed.

Regarding demographics, we observed significant Condition × Political Orientation interactions on all dependent measures: expertise-based trust, $B = -0.12$, $SE = 0.02$, $t(634) = -7.40$, $p < .001$, hypocrisy, $B = 0.17$, $SE = 0.02$, $t(634) = 10.44$, $p < .001$, credibility, $B = -0.27$, $SE = 0.03$, $t(634) = -9.50$, $p < .001$, and trust in the researchers' field, $B = -0.09$, $SE = 0.02$, $t(634) = -6.09$, $p < .001$. We also observed a significant Condition × Age interaction (three participants did not indicate their age) on credibility, $B = -0.02$, $SE = 0.01$, $t(631) = -2.86$, $p = .004$, but not on expertise-based trust, $B < 0.01$, $SE < 0.01$, $t(631) = 0.93$, $p = .354$, hypocrisy, $B = 0.01$, $SE < 0.01$, $t(631) = 1.58$, $p = .115$, and trust in the researchers' field, $B < -0.01$, $SE < 0.01$, $t(631) = -0.03$, $p = .980$. As in Study 1, we did not observe systematic interactions of participant gender (2 participants did not indicate their gender; unfortunately, too few non-binary [$n = 14$] and diverse [$n = 1$] people participated to be included in this analysis), target sex, or their combination with condition: hypocrisy, Condition × Participant Gender, F(1,

615) = 1.65, p = .199, $\eta^2_p < .01$, 90% CI [<.01, .01], Condition × Target Sex, $F(1, 615) = 0.20$, p = .657, $\eta^2_p < .01$, 90% CI [<.01, .01], Condition × Participant Gender × Target Sex, F(1, 615) = 1.31, p = .252, $\eta^2_p < .01$, 90% CI [<.01, .01], expertise-based trust, Condition × Participant Gender, $F(1, 615) = 0.09$, p = .763, $\eta^2_p < .01$, 90% CI [<.01, .01], Condition × Target Sex, $F(1, 615) = 3.51$, p = .062, $\eta^2_p < .01$, 90% CI [<.01, .02], Condition × Participant Gender × Target Sex, $F(1, 615) = 2.23$, p = .136, $\eta^2_p < .01$, 90% CI [<.01, .02], credibility, Condition × Participant Gender, $F(1, 615) = 1.80$, p = .180, $\eta^2_p < .01$, 90% CI [<.01, .01], Condition × Target Sex, $F(1, 615) = 1.28$, p = .259, $\eta^2_p < .01$, 90% CI [<.01, .01], Condition × Participant Gender × Target Sex, $F(1, 615) = 0.62$, p = .432, $\eta^2_p < .01$, 90% CI [<.01, .01], and trust in the researchers' field, Condition × Participant Gender, $F(1, 615) = 0.16$, p = .688, $\eta^2_p < .01$, 90% CI [<.01, .01], Condition × Target Sex, $F(1, 615) = 1.45$, p = .229, $\eta^2_p < .01$, 90% CI [<.01, .01], Condition × Participant Gender × Target Sex, $F(1, 615) = 2.43$, p = .119, $\eta^2_p < .01$, 90% CI [<.01, .02].

Supplementary Fig. 1

Johnson-Neyman analyses of significant moderation effects (Study 2).

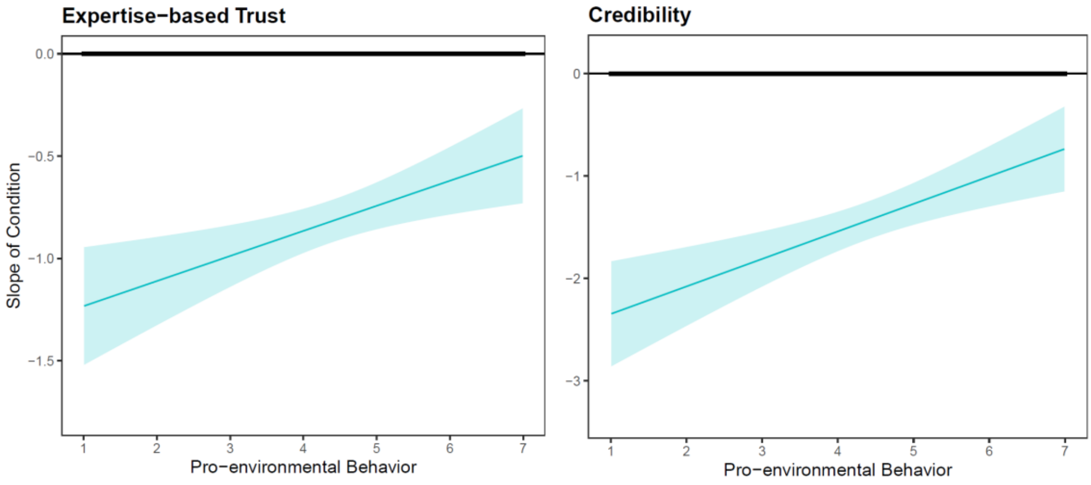

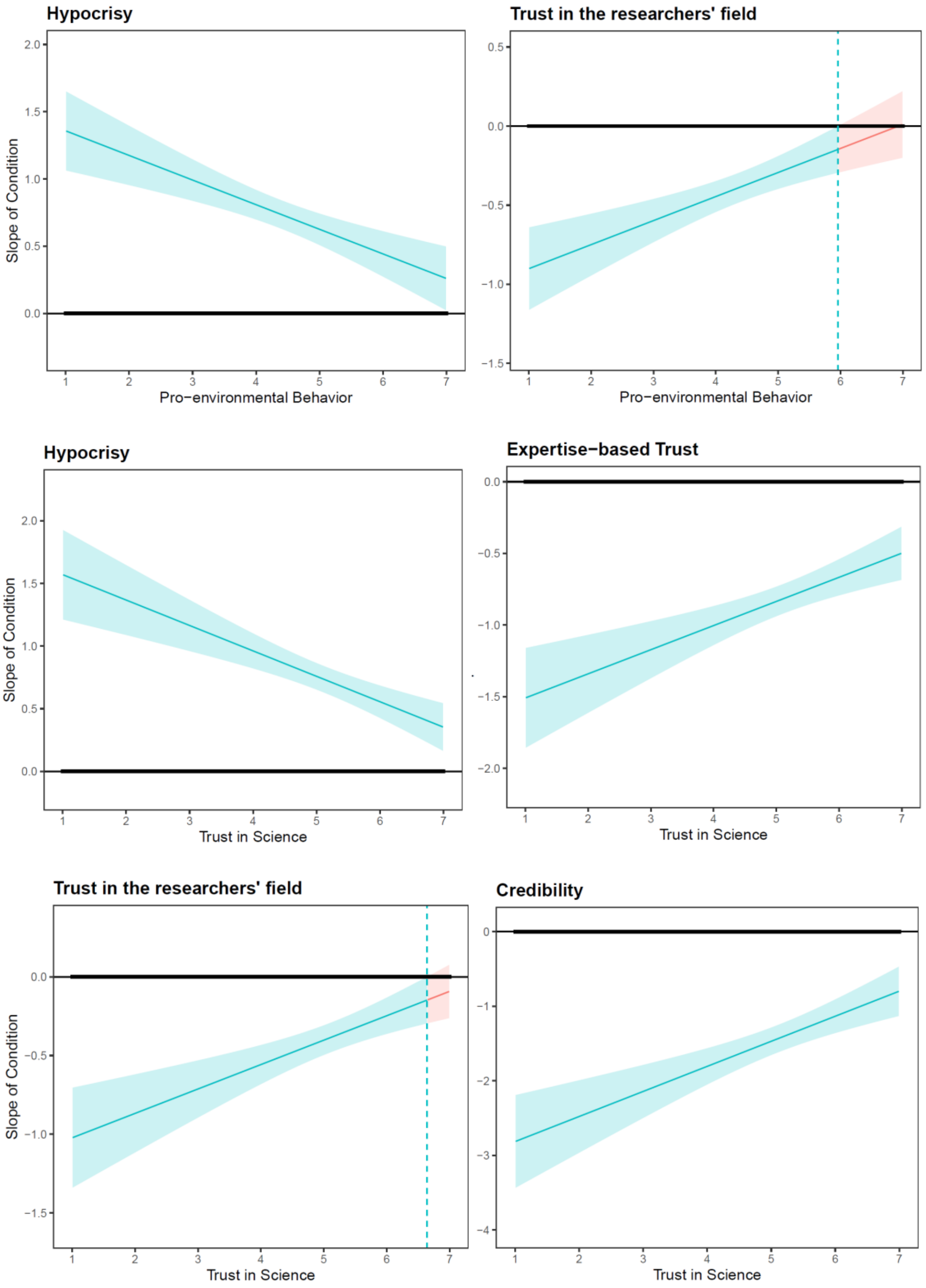
Hypocrisy
Slope of Condition
Pro−environmental Behavior
Trust in the researchers' field
Pro−environmental Behavior
Hypocrisy
Slope of Condition
Trust in Science
Expertise−based Trust
Trust in Science
Trust in the researchers' field
Slope of Condition
Trust in Science
Credibility
Trust in Science

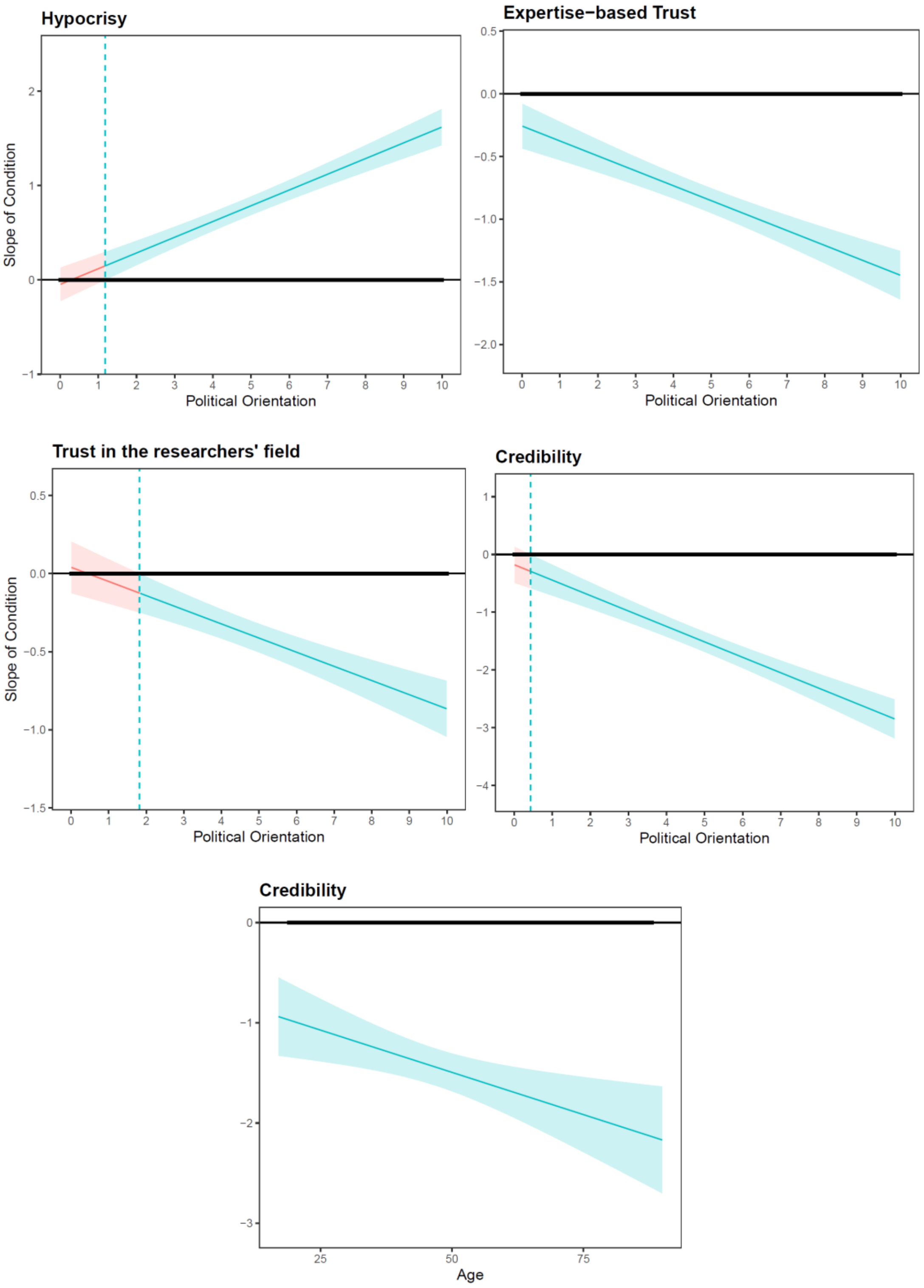

Note: The source activism effect (scientist activist vs. scientist non-activist) was not significant in red-shaded areas and significant at $p < .05$ in the green-shaded areas. The y-axis indicates the observed effect size of the activist vs. non-activist scientist manipulation. Values above 0 indicate that evaluation values on the dependent variable were greater for activists than non-activists; values below 0 indicate that values on the dependent variable were lower for activists than non-activists. Thick horizontal line indicates the range of observed values of the moderator; dashed green lines indicate transition points (i.e., moderator values where the condition effect transitions between significant and non-significant).

Johnson-Neyman analyses indicated that more left-leaning participants showed smaller negative effects of scientists' activism. Significant transition points emerged on hypocrisy (1.18), credibility (0.43) and the trust in the researchers' field (1.82). Older participants showed a larger condition effect on credibility, but no transition points were observed.

Including participants' self-description as an activist (yes vs. no) as an exploratory factor showed a significant Source Activism × Participant Activism interaction on hypocrisy, $F(1, 617) = 20.10, p < .001, \eta^2_p = .03$, 90% CI [.01, .06], expertise-based trust, $F(1, 617) = 13.62, p < .001, \eta^2_p = .02$, 90% CI [.01, .04], credibility, $F(1, 617) = 26.08, p < .001, \eta^2_p = .04$, 90% CI [.02, .07], and trust in the researchers' field, $F(1, 617) = 20.26, p < .001, \eta^2_p = .03$, 90% CI [.01, .06]. Follow-up analyses (Bonferroni-corrected) indicated a significant Source Activism effect among non-activist participants, but this effect did not emerge or was weaker among activist participants.

Turning to participants' self-description as a scientist (yes vs. no) as an exploratory factor, we observed no significant Source Activism × Participant Scientism interaction on hypocrisy, $F(1, 617) < 0.01, p = .998, \eta^2_p < .01$, 90% CI [<.01, <.01], expertise-based trust, $F(1, 617) = 4.03, p = .045, \eta^2_p < .01$, 90% CI [<.01, .02], credibility, $F(1, 617) = 0.27, p = .605, \eta^2_p < .01$, 90% CI [<.01, .01], and trust in the researchers' field, $F(1, 617) = 1.30, p = .255, \eta^2_p < .01$, 90% CI [<.01, .01].

References

1. R-Core-Team. R: A language and environment for statistical computing.). R Foundation for Statistical Computing (2020).

2. Revelle WR. psych: Procedures for personality and psychological research.). R package version 1.0.8 edn (2020).

3. Wickham H, Bryan J. *readxl: Read Excel Files*. R package version 1.4.3 (2023).

4. Wickham H. *stringr: Simple, Consistent Wrappers for Common String Operations*. R package version 1.5.1 (2023).

5. Wickham H, François R, Henry L, Müller K, Vaughan D. *dplyr: A Grammar of Data Manipulation*. R package version 1.1.4 (2023).

6. Wickham H. Reshaping Data with the reshape Package. *Journal of Statistical Software* **21**, 1 - 20 (2007).

7. Torchiano M. *effsize: Efficient Effect Size Computation*. R package version 0.8.1 (2020).

8. Long JA. *interactions: Comprehensive, User-Friendly Toolkit for Probing Interactions*. R package version 1.2.0 (2024).

9. Kuznetsova A, Brockhoff PB, Christensen RHB. lmerTest Package: Tests in Linear Mixed Effects Models. *Journal of Statistical Software* **82**, 26 (2017).

10. Long JA. jtools: Analysis and presentation of social scientific data. *Journal of Open Source Software* **9**, 6610 (2024).

11. Wickham H. ggplot2. *WIREs Computational Statistics* **3**, 180-185 (2011).

12. Auguie B. *gridExtra: Miscellaneous Functions for "Grid" Graphics*. R package version 2.3 (2017).

13. Murrell P. The gridGraphics package. *The R Journal* **7**, 151-162 (2015).

14. Wilke C. Streamlined Plot Theme and Plot Annotations for “ggplot2”[R Package Cowplot Version 1.1. 1].) (2020).

15. Robinson D. broom: An R package for converting statistical analysis objects into tidy data frames. *arXiv preprint arXiv:14123565*, (2014).

16. Bates D, Maechler M, Bolker B, Walker. S. Fitting linear mixed-effects models using lme4. *Journal of Statistical Software* **67**, 1-48 (2015).

17. Corporation M, Weston S. *doSNOW: Foreach Parallel Adaptor for the 'snow' Package*. R package version 1.0.20 (2022).

18. Microsoft, Weston S. *foreach: Provides Foreach Looping Construct*. R package version 1.5.2 (2022).

19. Singmann H, Bolker B, Westfall J, Aust F, Ben-Shachar M. Analysis of Factorial Experiments (afex), R package.). 1.5-0 edn (2025).

20. Ben-Shachar MS, Lüdecke D, Makowski D. effectsize: Estimation of effect size indices and standardized parameters. *Journal of Open Source Software* **5**, 2815 (2020).

21. Lenth RV. *emmeans: Estimated Marginal Means, aka Least-Squares Means*. R package version 1.10.4 (2024).
